# Marital status shows a strong protective effect on long-term mortality among first acute myocardial infarction-survivors with diagnosed hyperlipidemia – findings from the MONICA/KORA myocardial infarction registry

**DOI:** 10.1186/1471-2458-14-98

**Published:** 2014-01-30

**Authors:** Philip Andrew Quinones, Inge Kirchberger, Margit Heier, Bernhard Kuch, Ines Trentinaglia, Andreas Mielck, Annette Peters, Wolfgang von Scheidt, Christa Meisinger

**Affiliations:** 1 Institute of Epidemiology II, Helmholtz-Zentrum München, German Research Center for Environmental Health, Neuherberg, Germany; 2 KORA Myocardial Infarction Registry, Central Hospital of Augsburg, Augsburg, Germany; 3 Department of Internal Medicine I, Central Hospital of Augsburg, Augsburg, Germany; 4 Department of Internal Medicine/Cardiology, Hospital of Nördlingen, Nördlingen, Germany; 5 Institute of Health Economics and Health Care Management, Helmholtz-Zentrum München, German Research Center for Environmental Health, Neuherberg, Germany

**Keywords:** Infarction, Myocardial, Marital status, Mortality, Epidemiology, Follow-up studies

## Abstract

**Background:**

Reduction of long term mortality by marital status is well established in general populations. However, effects have been shown to change over time and differ considerably by cause of death. This study examined the effects of marital status on long term mortality after the first acute myocardial infarction.

**Methods:**

Data were retrieved from the population-based MONICA (Monitoring trends and determinants on cardiovascular diseases)/KORA (Cooperative Health Research in the Region of Augsburg)-myocardial infarction registry which assesses cases from the city of Augsburg and 2 adjacent districts located in southern Bavaria, Germany. A total of 3,766 men and women aged 28 to 74 years who were alive 28 days after their first myocardial infarction were included. Hazard ratios (HR) for the effects of marital status on mortality after one to 10 years of follow-up are presented.

**Results:**

The study population included 2,854 (75.8%) married individuals. During a median follow-up of 5.3 years, with an inter-quartile range of 3.3 to 7.6 years, 533 (14.15%) deaths occurred. Among married and unmarried individuals 388 (13.6%) and 145 (15.9%) deaths occurred, respectively. Overall marital status showed an insignificant protective HR of 0.76 (95% confidence interval (CI) 0.47-1.22). Stratified analyses revealed strong protective effects only among men and women younger than 60 who were diagnosed with hyperlipidemia. HRs ranged from 0.27 (95% CI 0.13-0.59) for a two-year survival to 0.43 (95% CI 0.27-0.68) for a 10-year survival. Substitution of marital status with co-habitation status confirmed the strata-specific effect [HR: 0.52 (95% CI 0.31-0.86)].

**Conclusions:**

Marital status has a strong protective effect among first myocardial infarction survivors with diagnosed hyperlipidemia, which diminishes with increasing age. Treatments, recommended lifestyle changes or other attributes specific to hyperlipidema may be underlying factors, mediated by the social support of spouses. Underlying causes should be examined in further studies.

## Background

Acute myocardial infarctions (AMI) and coronary heart disease (CHD) are the most common causes of death and the fourth largest contributor to the burden of disease worldwide [1,2]. The short-term mortality of AMI in industrialized countries is declining due to advances in treatment and prevention procedures [3-6]. Thus, closer examination of factors affecting long-term mortality among AMI survivors may deliver valuable knowledge for both clinical and public health practices. The protective effect of marital status and/or co-habitation on long-term mortality and other health outcomes has been well established in numerous studies among general populations [7-20]. However, studies examining trends in mortality have reported changes in the effects of marital status on mortality and self-rated health over the past decades [9,11,21]. Within the West German population, changes in gender roles and other social and cultural norms during the post-war era are made responsible for the changing impact of marriage or co-habitation on health and mortality over time [9]. Furthermore, a study conducted on the complete elderly Norwegian population reported that trends in the effects of marital status differ considerably between mortality causes [10]. Particularly large and increasing mortality differences between married and unmarried men and women have been observed for deaths resulting from circulatory diseases among Norwegians and for deaths resulting from AMI among Australians [10,21]. As a consequence, effects of marital status on health may not only be specific to time, culture and region, but may also depend on the disease. Generalizability of previous findings may consequently be questionable. Disease- and region-specific examinations are thus warranted. Unfortunately, only few studies have examined the effects of marital status or co-habitation on long-term mortality among first AMI survivors over the past 30 years [21-27]. The aim of our study was to examine the effect of marital status and co-habitation on one to 10-year mortality among first AMI survivors aged 28 to 74 years at the time of infarction. This study is the first to examine the effect of marital status on long-term survival of AMI survivors within a German population.

## Methods

The population-based Augsburg Myocardial Infarction Registry began continuously registering all cases of coronary deaths and non-fatal AMI in 1984 within the framework of the MONICA (Monitoring trends and determinants on cardiovascular diseases)-project. The registry has been part of the KORA (Cooperative Health Research in the Region of Augsburg) framework since 1995. The data covers the 25–74 year old population in the city of Augsburg and the two adjacent districts located in southern Bavaria, Germany (totalling 600,000 inhabitants). Patients hospitalised in 8 hospitals within the study region and 2 hospitals in the surrounding areas are included. Approximately 80% of all AMI cases in the study region are treated in the study region’s major hospital, “Klinikum Augsburg”, a tertiary care centre offering invasive and interventional cardiovascular procedures, as well as heart surgery facilities [28,29]. The study was approved by the Ethics Committee of the Bavarian Medical Association. All participants submitted written informed consent before being enrolled in the study. Methods of case identification, diagnostic classification of events, and data quality control have been described elsewhere [28,29].

### Sample

All patients registered between January 1, 2000, and December 31, 2008, who survived longer than 28 days after a first AMI were initially included into the sample. The follow-up was continued until August 26th 2010. Among 4,405 men and women who survived 28 days after their first AMI, we excluded 298 patients without information on marital status. Additionally, 341 individuals with incomplete covariate data were excluded. The final sample size was 3,766 persons aged 28 to 74 years.

### Data collection and endpoints

Study participants were interviewed with a standardised questionnaire during their hospital stay after being transferred from the intensive care unit. The interviews were performed by trained study nurses and covered demographic information, risk factors and co-morbidities. Marital status and living arrangements were assessed via interview, and dichotomized as married or unmarried, and living alone or living together with someone, respectively. Applied and recommended compositions of marital status and co-habitation as predictors of long-term disease and mortality outcomes are heterogeneous throughout the literature [7,14-16,18,26]. Consequently, we let the most suitable explanatory variable composition be defined by the properties of the available data. Socio-economic status (SES) was assessed as the minimal years of schooling required for the highest school degree attained. Based on the German educational system, nine years was defined as low SES while more than nine years was defined as 'higher SES’. Risk factors and co-morbidities were determined either by patient report (smoking) or by chart review (e.g. hyperlipidemia, diabetes, stroke). Information on AMI characteristics and treatment (e.g. AMI type, type of reperfusion therapy) as well as in-hospital complications (e.g. cardiac arrest, pulmonary oedema) were also determined by chart review.

The study’s end point was long-term all-cause mortality among patients after their first AMI. Mortality was assessed by checking the vital status of all registered persons in the KORA AMI registry through the population registries, inside and outside the study area in 2010. This procedure guaranteed that the vital status of cohort members who had moved outside of the study area could also be assessed. Death certificates were obtained from local health departments.

### Statistical analyses

Relevant explanatory variables were determined through a literature search and grouped into: socio-demographic factors, patient history, behavioural factors, clinical parameters, treatment and in-hospital complications [14-16,19,30-46]. After initial descriptive analyses, all explanatory variables were subjected to bivariate Log-Rank tests against 10.7-year survival. Variables which were significant at an alpha-level of 0.2 were included into a multivariate Cox regression model fitting. Marital status was the primary, binary, explanatory variable of interest. Co-habitation was examined in sensitivity analyses. Variation in the time of study entry was adjusted for by inclusion of the variable “recruitment day”. Recruitment day was defined as the number of consecutive days between December 31, 2000 and the recruitment date. Initial model fitting was concluded via manual backward elimination using a sample of n = 3,398 complete cases. Marital status, sex, age-group and recruitment day were forced into the model. Based on the results of bivariate Log-Rank tests, SES, hypertension, angina pectoris, hyperlipidemia, stroke, diabetes, obesity, ST-elevation myocardial infarction, bundle-branch-block, cardiac arrest, pulmonary oedema, and reperfusion therapy were introduced into the model fitting procedure. Among these, obesity, SES, and ST-elevation were removed from the model as they failed to show significant effects on 10.7-year survival. Finalized model fitting was computed using the maximal available sample of n = 3,766 after omitting SES which reduced missing values by 368. Finalized model fitting yielded results identical to the initial analyses. The proportional hazards assumption was examined in the full model using the correlation of Schönfeld residuals against observation-time and squared observation-time for each explanatory variable, respectively. Violations of the proportional hazards assumption were observed for reperfusion therapy and hypertension. Interaction terms with observation time showed p-values of <0.0001 and 0.0154 respectively. Time-dependencies were incorporated into the model by introducing significant interaction terms. Multicollinearity in the covariate structure of the fully adjusted model was ruled out as variance inflation factors were below 1.2 for all explanatory variables. All explanatory variables in the full model were tested for interaction with marital status. Both minimally and fully adjusted models were run as analyses stratified by variables which significantly interacted with marital status (age-group, hyperlipidemia). In a final step, fully adjusted, stratified models were run for different survival cut-offs from one to ten years. All tests within the multivariate model fitting were conducted at an alpha level of 0.05.

**Figure 1:**
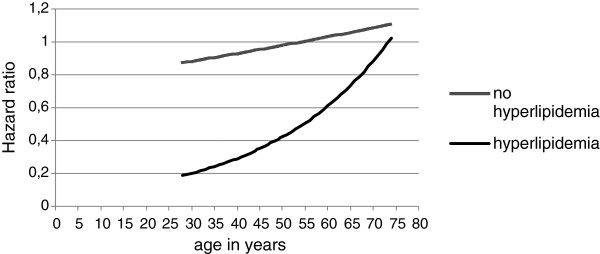
Associations between age in years and the effect of marital status on 10.7-year survival – fully adjusted model stratified by hyperlipidemia.

Several sensitivity analyses were performed. First, a model without the information loss produced by dichotomization of the continuous variable age was compared with the main analyses. Second, the fully adjusted model was rerun stratified only by hyperlipidemia with age introduced as a continuous variable and the inclusion of an interaction term between age and marriage, as shown in Figure 1. Third, since a combination of marital status with co-habitation produced strata with too infrequent events for stable multivariate analyses, the stratified, fully adjusted model was rerun with co-habitation instead of marital status as the main explanatory variable to confirm any observed associations.

All Cox modelling procedures were performed with the exact method assuming an existing order in tied measurements. All statistical analyses were performed using SAS software, release 9.2 (SAS Institute, Cary, NC).

## Results

Within the total study population, 2,854 (75.8%) individuals were married and 912 (24.2%) were unmarried. Among married individuals, 2,795 (97.9%) were living together. Among the 3,129 individuals living together with someone, only 334 (10.7%) were unmarried. Being married was more common among men 2,283 (80.4%) than women 571 (61.7%). The median age was 62 years with an interquartile range of 53 to 68 years. Marital status did not differ between age groups. Within the age-group 28–59 years, 1,177 (73.8%) individuals were married, while 1,677 (77.2%) individuals were married among those aged 60–74 years.

**Figure 2:**
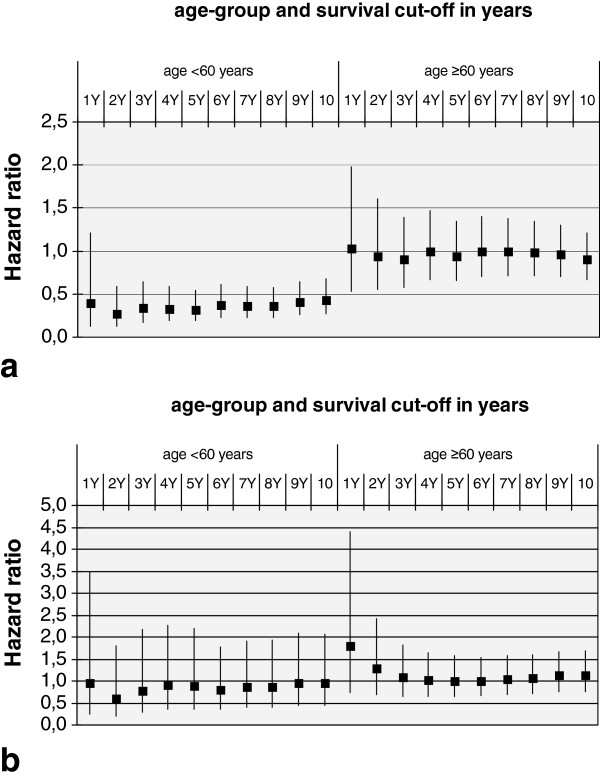
**Effect of marital status on one- to ten-year survival stratified by age and hyperlipidemia. a**. Hazard ratios with 95% confidence limits among individuals with documented hyperlipidemia. **b**. Hazard ratios with 95% confidence limits among individuals without documented hyperlipidemia.

**Figure 3:**
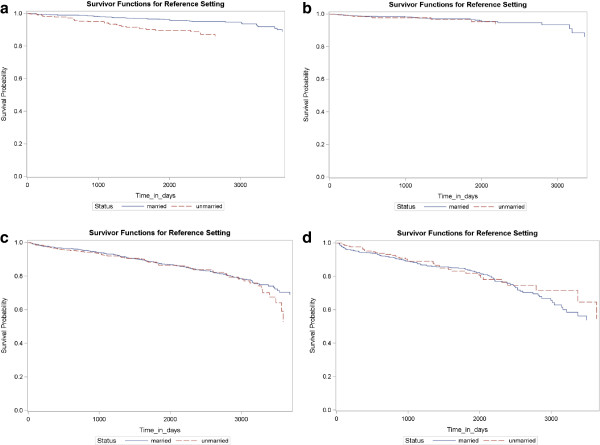
**Kaplan-Meier estimates for 10.7 year survival stratified by age and hyperlipidemia. a**. age < 60 years, hyperlipidemia, n = 1136. **b**. age < 60 years, no hyperlipidemia, n = 458. **c**. age ≥ 60 years, hyperlipidemia, n = 1487. **d**. age ≥ 60 years, no hyperlipidemia, n = 685.

During a median follow-up of 5.3 years with an inter-quartile range of 3.3 to 6.6 years, observation times ranged from 32 to 3889 days (10.7 years). A total of 533 (14.2%) deaths were recorded. Among married individuals, 388 (13.6%) deaths occurred. Among unmarried individuals 145 (15.9%) deaths were observed. Further details of descriptive and explorative analyses are presented in Table 1. Interaction terms between marital status and all other explanatory variables were only significant for hyperlipidemia and age-group (0.0451 and 0.0346 respectively). The age-group and hyperlipidema stratified analyses displayed in Models 3, 4, and 5 of Table 2 and Figures 2 and 3 showed a strong, and highly significant protective effect of marital status only among men and women aged 28–59 with hyperlipidemia. Adjustment of the stratified model for relevant covariates did not change the effect estimates for marital status, as comparisons between the Models 3, 4 and 5 of Table 2 show. The analyses displayed in Figure 2 demonstrate the consistency of a strata-specific effect of marital status over all examined observation times, ranging from two to 10 years, for individuals aged 28–59 years with hyperlipidemia. Effect estimates range from a hazard ratio (HR) of 0.27 (95% confidence interval (CI) 0.13-0.59) for a two-year survival to a HR of 0.43 (95% CI 0.27-0.68) for a 10-year survival. In the other three strata, no significant effects were observed. Among the survival times displayed in Figure 2, effect estimates for marital status varied for the one year survival cut-off. The low event frequency for this survival time may have been insufficient to allow the calculation of precise estimates.

**Table 1:** MONICA/KORA acute myocardial infarction registry, 3,766 AMI survivors age 25 to 74 years

**Variable**	**Category**	**Observations n (%)**	**Events n (%)**	**LogRank Chi**^**2**^^**a**^	**p-value**^**a**^
**Sociodemographic factors**					
Married	Yes	2854 (75.78)	388 (13.59)		
	No	912 (24.22)	145 (15.90)	5.3761	0.0202
Living together	Yes	3129 (83.09)	434 (13.87)		
with someone	No	637 (16.91)	99 (15.54)	2.0646	0.1508
Age-group	28-59	1594 (42.33)	116 (7.28)		
	60-74	2172 (57.67)	417 (19.20)	113.2626	<0.0001
Sex	Female	926 (24.59)	149 (16.09)		
	Male	2840 (75.41)	384 (13.52)	3.8670	0.0492
SES^b,^^g^	Low	2488 (73.22)	350 (14.07)		
	High	910 (26.78)	91 (10.00)	6.1249	0.0133
**Patient history**					
Hypertension	Yes	2859 (75.92)	440 (15.39)		
	No	907 (24.08)	93 (10.25)	21.4460	<0.0001
Angina pectoris	Yes	538 (14.29)	117 (21.75)		
	No	3228 (85.71)	416 (12.89)	28.6078	<0.0001
Hyperlipidemia	Yes	2623 (69.65)	340 (12.96)		
	No	1143 (30.35)	193 (16.89)	29.4056	<0.0001
Stroke	Yes	239 (6.35)	81 (22.89)		
	No	3527 (93.65)	452 (12.81)	80.7935	<0.0001
Diabetes	Yes	1076 (28.57)	219 (20.35)		
	No	2690 (71.43)	314 (11.67)	47.7729	<0.0001
**Behavioural factors**					
Ever smoker^g^	Yes	2482 (68.94)	320 (12.89)		
	No	1118 (31.06)	155 (13.86)	0.0336	0.8545
Body mass index	Yes	947 (25.15)	112 (11.83)		
≥ 30 Kg/m^2^	No	2819 (74.85)	421 (14.93)	3.2079	0.0733
**Clinical parameters, treatment, in hospital complications**					
ECG^c^					
ST-elevation	Yes	1484 (39.41)	202 (13.61)		
	No	2282 (60.59)	331 (14.50)	5.2685	0.0217
Bundle-branch-block	Yes	194 (5.15)	51 (26.29)		
	No	3572 (94.85)	482 (13.49)	28.5613	<0.0001
Reperfusion therapy	Yes	3191 (84.73)	357 (11.19)		
	No	575 (15.27)	176 (30.61)	138.5795	<0.0001
PTCA^d^	Yes	2553 (67.79)	254 (9.95)		
	No	1213 (32.21)	279 (23.00)	73.2404	<0.0001
CABG^e^	Yes	593 (15.75)	87 (14.67)		
	No	3173 (84.25)	446 (14.06)	<0.0001	0.9972
Other^f^	Yes	110 (2.92)	24 (21.82)		
	No	3656 (97.08)	509 (13.92)	0.4325	0.5108
Cardiac arrest	Yes	242 (6.43)	51 (21.07)		
	No	3524 (93.57)	482 (13.68)	7.5719	0.0059
Pulmonary edema	Yes	82 (2.18)	32 (39.02)		
	No	3684 (97.82)	501 (13.60)	50.7943	<0.0001
Reinfarction^g^	Yes	80 (2.13)	12 (26.25)		
	No	3684 (97.87)	521 (14.14)	0.0329	0.8561

^a^Bi-variate, unadjusted LogRank-tests of effects on 10.7-year mortality.

^b^Socioeconomic status.

^c^Electrocardiogram.

^d^Percutaneous Transluminal Coronary Angioplasty.

^e^Coronary Artery Bypass Graft.

^f^Percutaneous Coronary Intervention without stent, Thrombolysis.

^g^All bi-variate tests shown were applied to the maximal available sample of 3766 complete cases, which was utilized in the final regression models. Some of the variables, which were omitted before or during model fitting, have missing values. Data is missing for 368 individuals for SES, 166 individuals for ever smoker and 2 individuals for reinfarction.

**Table 2:** Effect of marital status on 10.7-year mortality

	**Stratification**					
**Model**	**Age group**	**Hyperlipidemia known**	**N**	**Wald p-value**	**Hazard ratio**	**Confidence interval 95%**
Model 1^a^	-	-	3766	0.0207	0.80	0.66-0.97
Model 2^b^	-	-	3766	0.0688	0.83	0.68-1.01
Model 3^c^	<60	Yes	1136	0.0003	0.44	0.28-0.69
	≥60	Yes	1487	0.0603	0.77	0.58-1.01
	<60	No	458	0.8193	1.09	0.52-2.27
	≥60	No	685	0.6097	1.10	0.76-1.59
Model 4^d^	<60	Yes	1136	0.0003	0.44	0.28-0.69
	≥60	Yes	1487	0.1387	0.80	0.60-1.08
	<60	No	458	0.8067	1.10	0.52-2.30
	≥60	No	685	0.7002	1.08	0.74-1.58
Model 5^e^	<60	Yes	1136	0.0002	0.43	0.27-0.67
	≥60	Yes	1487	0.4926	0.90	0.67-1.21
	<60	No	458	0.9053	0.95	0.44-2.08
	≥60	No	685	0.6264	1.10	0.75-1.63

^a^Model 1: unstratified, unadjusted;

^b^Model 2: unstratified, adjusted for sex, age ≥60, recruitment day, reperfusion therapy, hyperlipidemia, angina pectoris, diabetes, stroke, hypertension, bundle branch block, pulmonary edema, cardiac arrest;

^c^Model 3: stratified for age and hyperlipidemia, unadjusted;

^d^Model 4: stratified for age and hyperlipidemia, adjusted for sex and recruitment day;

^e^Model 5: stratified for age and hyperlipidemia, adjusted for sex, recruitment day, reperfusion therapy, angina pectoris, diabetes, stroke, hypertension, bundle branch block, pulmonary edema and, cardiac arrest.

Results of the hyperlipidemia stratified, fully adjusted model with age in years introduced as a continuous variable, are presented in Figure 1. The two HR curves visualize how the effect of marital status on 10.7-year survival changes with increasing age in years for patients with and without documented hyperlipidemia. Each curve displays effect estimates combined from age and marital status. When the variable marital status was substituted with co-habitation the same strata-specific effect was observed. Within the strata of individuals aged 28–59 with hyperlipidemia, co-habitation had a HR of 0.52 (95% CI 0.31-0.86) in the fully adjusted model. Within the remaining strata HRs of 1.04 (95% CI 0.74-1.46) among those aged 60–74 with hyperlipidemia, 1.04 (95% CI 0.40-2.54) among those aged 28–59 without hyperlipidemia and 1.10 (95% CI 0.72-1.69) among those aged 60–74 without hyperlipidemia were observed. For comparison the results of the identical model conducted with marital status are displayed in Model 5 of Table 2.

## Discussion

The most dominant and relevant finding of this study is the observed effect modification of marital status by hyperlipidemia and by age. A strong protective effect of marriage on long-term survival was apparent among younger men and women with hyperlipidema. The protective effect decreased with increasing age and was much less pronounced and statistically insignificant among individuals without hyperlipidemia. The described strata-specific effect of marital status on survival was similar for the minimally adjusted and the fully adjusted models and was consistent over survival times from two to 10.7 years, and in the sensitivity analyses described above.

Our findings are consistent with previous studies from other countries which have examined all-cause mortality among AMI-survivors: a protective effect of marital status or co-habitation or a hazardous effect of being single, divorced or widowed on long-term survival has been reported [22-26,40]. In the study by Buchholz et al., the hazardous effect of living alone on 4-year survival (HR 1.35, 95% CI 0.94-1.93) was insignificant [24]. These studies were however, somewhat heterogeneous, as they differed considerably in their definitions of marital status or co-habitation, sample sizes, countries, regions and investigated timespans, which ranged from 1967–1971 to 2008–2009 [22,26]. Finally, these studies also differed in their follow-up periods which ranged from one year [26] to 16 years [23]. In our study effects were visible for men and women. Differences between males and females observed in the general West German population by Brockmann and Klein were not pronounced enough to be significant in our sample [9]. Our results confirm the findings of several studies which observed protective effects of marriage or co-habitation on long-term survival for both men and women [7,22,23,26,27]. The fact that diagnosed hyperlipidemia is the only risk factor among all those considered, with a highly relevant and significant protective effect on long-term survival, deserves special attention. In the full, unstratified model without interaction terms hyperlipidemia had a HR of 0.64 (95% CI 0.53-0.76). The protective effect of diagnosed hyperlipidemia on long-term all-cause mortality observed in our study confirms previous findings from Spain [40]. Nutritional and pharmacological treatment regimes, specific to patients with hyperlipidemia, such as diets and statins, are known to reduce the hazard of cardiovascular events such as stroke and recurrent AMI in high risk patients and thus should also reduce mortality [47-52].

Our findings should be considered in the context of established theoretical framework. Theories regarding the causality behind marital status and mortality have been roughly grouped into three types: selection, social causation and stress theories [9-11,19,20,53,54]. Selection theories assume that individuals with poor health are less likely to both establish and maintain long-term relationships, such as marriages. Thus, health status affects marital status as well as mortality. This represents a concern when interpreting the current findings as it implies the potential existence of reverse causation [9,10,19]. Social causation theories propose that married individuals draw health benefits from spousal support with regards to seeking of treatment, adherence to treatment and recommended lifestyle changes, as well as greater financial resources, which make medical treatment and healthy lifestyle choices affordable [9-11,19,20,54]. Stress-related theories propose that the effects of partner loss or quality of the relationship may affect health and thus survival [9-11,19]. Finally, a specific so far unnamed theory, which we here describe as the behavioural assimilation theory, can be derived from the results of a Japanese study [53]. It reported that co-habitating, married couples adopt similar healthy as well as unhealthy behavioural patterns. Within the context and setting of our main findings, the social causation theory enables the generation of very plausible hypotheses. We assume married men and women to be more adherent to medical treatment and/or dietary regimes, due to spousal support and shared financial resources. Thus, the beneficial effects of treatment and/or diet have greater opportunity to affect individuals with diagnosed hyperlipidemia. This theory confirms the results of a study by Wu et al. which examined the effect of marital status on the adherence to medical regimes in 136 patients with heart failure [55]. Modification of treatment effects by marital status may provide a partial explanation for the differences in the effects observed between diseases among the elderly in Norway [10].

The reduction of the protective effect of marital status on mortality with increasing age is expected and confirms findings of several studies, including a study of all-cause mortality in the West German population by Brockmann & Klein. The latter study observed a change in the effect of marital status on all-cause mortality with age. In women, single status advanced from the state with the highest risk to the state with the lowest risk with increasing age. In men the same effect was overestimated, as single men were very infrequent at higher ages [9]. A US study of all-cause mortality and a Finnish study of both all-cause and cardiovascular mortality showed a similar deterioration of the protective effect of marital status with increasing age [7,16]. However, the underlying causalities remain to be determined here as well, and may include several underlying factors. Health-related behaviour among married couples may be specific to generations due to social, economic and cultural developments [9]. In younger generations increasing public health promotion and resulting changes in public awareness and trends in lifestyle may also have important impacts. Finally, spousal support and perceived stress in relationships may also differ depending on the duration of marriage [9].

The replacement of marital status with co-habitation yielded the same strata-specific protective effect. The effect was slightly less pronounced for the co-habitation variable, due to a reduced protective effect in the unmarried, co-habitating men and women in our sample. These findings confirm the results of several studies, which show considerably lower risks of death from both all-cause and cardiovascular deaths among married couples living together when compared to unmarried co-habitating individuals, widows, and individuals living alone [7,16,18,26]. A meta-analyses conducted by Rendall et al. found little evidence of differences between above differentiations of unmarried individuals [7]. In contrast to our findings, two studies reported a more pronounced effect of cohabitation on long-term all-cause mortality [14,15].

### Strengths and limitations

Our study is the first to examine the relationship between marital status and mortality among a group of first AMI survivors within a German population. Due to the longitudinal design and consistency of the results across several different analyses, our study offers strong evidence for a disease-specific mediation of treatment effects on long-term mortality by marriage. The observed differences between strata are very strong and distinct. The results are coherent with theoretical background, plausible, and consistent with previous studies. They furthermore enable the derivation of a new hypothesis, which suggests that future analyses should examine treatment effects specific to hyperlipidemia.

However, a set of limitations must be considered. A total of 639 individuals constituting 14.5% of the total sample had to be excluded due to missing values in explanatory variables. An examination of the effects of marital status on different death causes was not possible within our population but should be pursued in further research. Furthermore, in explorative analyses 12 interaction terms were tested in order to identify potential interactions with marital status. P-values were not adjusted for multiple comparisons due to low statistical power. Studies on different populations will be required to replicate and confirm the effects reported here. Explanatory variables were measured once at baseline. Thus, we were unable to consider the changes of explanatory variables over time. As the co-morbidity variable “diagnosed hyperlipidemia” was combined using information collected before and during hospitalization, the applied diagnostic criteria lacked standardization. Finally, data on men and women older than 74 years were not available for our sample.

## Conclusions

Marital status appears to have a strong protective effect in first AMI-survivors with diagnosed hyperlipidemia. This effect is most pronounced in young adults and diminishes with increasing age. We derive the hypotheses that treatments, recommended lifestyle changes or other attributes specific to hyperlipidema may be underlying factors that are mediated by the social support of spouses. More generally, differences in the effects of marital status on long-term mortality from different diseases may be partially caused by treatments and behaviours, which are mediated by marriage. Underlying causal factors should be examined in future studies.

## Abbreviations

AMI: Acute myocardial infarction; CHD: Coronary heart disease; CI: Confidence interval; HR: Hazard ratio; KORA: Cooperative Health Research in the Region of Augsburg; MONICA: Monitoring trends and determinants on cardiovascular diseases; SES: Socio-economic status.

## Competing interests

The authors declare that they have no competing interests.

## Authors’ contributions

PAQ developed the study question, performed all data analyses and drafted the manuscript. IT prepared the data sets performed plausibility checks and variable transformations. CM, BK, WVS, AP and MH developed, organized and operated the complete MONICA-KORA registry and data assessment. AM was advisor for sociodemographic and behavioural determinants of health. CM and IK were general counselors and advisors for data analyses and manuscript preperation. All authors reviewed and revised preliminary manuscript drafts and approved the final manuscript.

## Pre-publication history

The pre-publication history for this paper can be accessed here:

http://www.biomedcentral.com/1471-2458/14/98/prepub
